# Moebius syndrome: clinical features, diagnosis, management and early intervention

**DOI:** 10.1186/s13052-016-0256-5

**Published:** 2016-06-03

**Authors:** Odoardo Picciolini, Matteo Porro, Elisa Cattaneo, Silvia Castelletti, Giuseppe Masera, Fabio Mosca, Maria Francesca Bedeschi

**Affiliations:** NICU, Department of Clinical Sciences and Community Health, Fondazione IRCCS Ca’ Granda Ospedale Maggiore Policlinico, Università degli Studi di Milano, Milan, Italy; Medical Genetic Unit, Fondazione IRCCS Ca’ Granda Ospedale Maggiore Policlinico, Milan, Italy; Scientific Committee of the Italian Moebius Syndrome Association, Muggiò, Milan, Italy; Pediatric Rehabilitation Unit, Fondazione IRCCS Ca’ Granda Ospedale Maggiore Policlinico, Via Manfredo Fanti 6, 20122 Milan, Italy

**Keywords:** Moebius syndrome, Cranial nerve, Facial paralysis, Abducens paralysis

## Abstract

**Background:**

Moebius syndrome (MBS) is rare disease characterized by nonprogressive congenital uni- or bi-lateral facial (i. e. VII cranial nerve) and abducens (i. e. VI cranial nerve) palsy. Although the neurological and ophthalmological findings are quite well-known, data concerning the attendant functional difficulties and their changes over time are seldom addressed.

In this study we attempt to estimate the prevalence of clinical and functional data in an Italian cohort affected by MBS.

**Methods:**

The study included 50 children, 21 males and 29 females, aged 1 month to 14 years. The patients entered into a multidisciplinary diagnostic and follow-up protocol that had the specific purpose of detecting clinical and developmental deficits related to MBS.

**Results:**

Involvement of the VII cranial nerve (total/partial, bilateral or unilateral) was present in 96 % of patients, and of the VI nerve in 85 %. Two patients were without impairment of the VII nerve and seven patients had no involvement of the VI nerve and were thus classified as Moebius-like because of the involvement of other CNs. Additional affected CNs were numbers III-IV in 16 %, V in 11 %, VIII and X each in 8 %, the XI in 6 %, the IX, most often partially, in 22 %, and the XII in 48 % of cases. Their development was characterized by global delay at one year of age, motor, emotional and speech difficulties at two years of age, a trend toward normalization at three years of age but with weakness in hand-eye coordination, and achieving average results at five years of age. Overall 90 % of children had a normal developmental quotient whereas only 10 % manifested cognitive deficits.

**Conclusion:**

Early rehabilitation may enhance the recovery of normal function, particularly in vulnerable areas of development. It is possible that early intervention that integrates sensory and visual information with emotional difficulties can improve the prognosis of the child with MBS.

## Background

Moebius syndrome (MBS) is a rare disease characterized by unilateral or bilateral nonprogressive congenital facial palsy (VII cranial nerve) with impairments of ocular abduction (VI cranial nerve); it can also be associated with other cranial nerve (CN) palsies, orofacial anomalies and limb defects [1]. This condition was originally described by Von Graefe in 1880 and by Moebius in 1888. Since then, more than 300 cases have been described and reported in the literature by a number of authors [2–8].

The prevalence of MBS is estimated to be 1/250.000 live births with equal incidence in both sexes. Most cases are sporadic, but familial cases, representing about 2 % of all affected individuals, have been documented.

The diagnosis of MBS is based exclusively on clinical criteria, although recent studies are beginning to document causative genetic patterns [9]. The lack of diagnostic criteria complicates the clinical assessment, definition of prognosis, and genetic analysis of patients with MBS. To address this concern, a group of clinicians and researchers met in 2007 at the biannual research meeting of Moebius Syndrome Foundation in Bethesda (MD USA) and defined the MBS as “congenital, uni- or bilateral, nonprogressive facial weakness and limited abduction of the eye(s)” [3].

The cause and pathogenesis of MBS remain unclear and controversial since the initial descriptions by von Graefe and Moebius, and there has been a decade long debate whether MBS has a genetic aetiology or not. Fetal toxic exposure, genetically determined vascular rhombencephalic disturbances in development, or an acquired ischemic event occurring after the fifth week of pregnancy have been proposed as determinants.

It has been supposed that a primary insult leads to a sequence of events involving one or more focal areas of damage perhaps in the brainstem where the neurons of the facial, abducens, and lacrimal (salivary) nuclei, are anatomically coincident during this phase of the embryogenesis.

On the other hand, de novo *PLXND1* and *REV3L* mutations have recently been identified in a a number of of MBS patients. However, *PLXND1* and *REV3L* represent totally unrelated pathways involved, respectively, neural migration during hindbrain development, and DNA translesion synthesis, essential for the replication of endogenously damaged DNA [9].

MBS can be recognized and diagnosed early during the neonatal period. Poor or absent sucking due to incomplete closure of the lips, lack of facial mimicking (especially while crying), fixed gaze, incomplete eyelid closure during sleep and ptosis have all been observed. Hypotonia and developmental delay can also be present.

Paralysis of the VII CN, is responsible for the absence of mimicry, the lack of smile and the suction deficit. Three specific patterns of ocular motility alterations have been identified [10]: pattern A consists of orthotropia in the primary position with a complete defect in both abduction and adduction ocular movements, found in 41 % of cases; pattern B, with large-angle esotropia (convergent strabismus), crossed fixation, documented in 50 % of cases; and pattern C, characterized by a large-angle exotropia (divergent strabismus) with torticollis, absence of convergence, and vertical eye misalignment with involvement of the III and IV CNs, seen in a minority of patients (9 %).

In a few patients other CNs can be compromised including paralysis of the XII CN giving rise to lingual palsy and hypoplasia; damage of the V CN affecting endo- and perioral sensitivity while damage to the IX CN impairs palatine and pharyngeal motility.

Impairment of the cochlear branch of the VIII CN can contribute to language delay [10–13].

Among MBS’s patients club foot, hand anomalies (syndactyly, brachydactyly, ectrodactyly) and agenesis of the pectoral muscle and dysmorphisms are occasionally observed. Association with other syndromes like Poland syndrome, Pierre Robin sequence, Carey-Fineman-Ziter, Klippel-Feil anomaly has also been reported [4].

In 1998 Abramson first proposed the acronym CLUFT: C, cranial nerve, L, lower limb; U, upper limb, F, face, T, thorax to define grading of disease, to describe the heterogeneity of the clinical features and establish a level of impairment (Table 1) [4].

**Table 1:** CLUFT: Grading system of Abramson et al. [4]

CLUFT Clinical Features	Grading
C: Cranial nerves	
VII nerve partial	0
VI nerve partial	1
VI e VII nerve complete	2
Additional nerve involvement	3
If bilateral and equal add	*B*
L: Lower extremity	
Normal	0
Talipes equinovarus, syndactyly, ankylosis	1
Absent phalanges	2
Longitudinal or transverse defect	3
U: Upper extremity	
Normal	0
Digital hypoplasia or failure of differentiation	1
Ectrodactyly	2
Failure of longitudinal or transverse formation	3
F: Facial structural anomalies	
Normal	0
Cleft palate	1
Micrognathia	2
Microtia, microphtalmia, abnormal joint etc.	3
T: Thorax	
Normal	0
Scoliosis	1
Pectoral hypoplasia or breast anomaly	2
Chest wall deformity, breast or pectoral aplasia	3
Total Score	

## Methods

### Setting

The Centre Moebius (CMM) in Fondazione IRCCS Ca’ Granda, Milan (The Center for the diagnosis and treatment of Moebius syndrome) was established in June 2003 as one of the programs of the Italian Moebius Syndrome Association Onlus (AISMo Onlus), aimed at developing referral centers for early diagnosis. The goals of the Center are: early identification of developmental abnormalities in newborns and infants with suspected MBS; supporting the parents with diagnostic, educational and rehabilitative interventions; creating a network with specialized services and personalized rehabilitation; disseminating knowledge about the neurobehavioral characteristics of the MBS child, for pediatricians and rehabilitation professionals; investigating the psychomotor and functional pathways of MBS subjects.

### Study Population

We report the clinical and functional analysis of a group of 50 Italian children (mean age 38 months, range 1 month to 14 years) with a suspected diagnosis of MBS, recruited from the AISMo Onlus, from the MBS Referral Center of the Hospital of Parma and from individual pediatricians [14–16]. To diagnose MBS, the major criteria developed by the the First Scientific Conference of Moebius Syndrome in 2007 were used, i.e., the presence of a congenital unilateral or bilateral nonprogressive paresis of the sixth and/or the seventh CNs. Children with additional involvement of other CNs and/or, motor, musculoskeletal and neurodevelopmental disorders were also included [10].

Patients who did not meet both major criteria were classified as having a Moebius-like syndrome and were considered separately.

### Statement regarding ethics committee approval

The study focuses on the observational description of patients followed in the period between 2003 and 2015. Patients underwent a protocol of assessments, which were normally administered to all children referred to the outpatients department. Data were obtained from clinical files and were retrospectively reported. Ethics committee approval is not required in Italy for this type of reports.

### Clinical evaluation and intervention

All MBS children were followed by a series of clinical and functional evaluations, as listed in Table 2. The multidisciplinary diagnostic and follow-up protocol included interviews with parents and caregivers (medical and genetic history), and physiatrist assessment to define the skills and functional development of the child for prognostic purposes and intervention. Patient care includes not only the child but takes into account the expectations and strengths of the family. Outpatient service provides physical rehabilitation for the movement disorders; feeding and speech therapy for the oral-motor functions deficits; psychomotor intervention because of communication difficulties and visuomotor coordination.

**Table 2:** Clinical examinations performed in the MBS Center

	Diagnosis	Follow up
Clinical genetic evaluation^b^	X	X
Physiatric evaluation^b^	X	X
Physiotherapy assessment		X
Oral-motor assessment		X
Psychological counseling^a^		X

^a^Follow up if necessary

^b^Second visit (within one month), Third visit (after 3–6 months)

Physical therapy, speech therapy, psychomotor treatments sessions are held from one to three times a week, depending on whether functions are merely delayed or can be improved by treatment. Psychological assistance was also offered to parents if needed.

Other instrumental and specific evaluations as reported in Table 3 were carried out by other Departments of the same institution. Psychomotor evaluation was formally assessed at 1, 2, 3 and 5 years of age according to the Griffiths Mental Development Scale Revised (GMDS-R) [17].

**Table 3:** Instrumental and clinical evaluation performed in other hospital departments

	Diagnosis	Follow up
Ophtalmological evaluation		X
Neurological evaluation		X
Brain and NMR	X	
Audiological evaluation^a^	X	
Orthopedic evaluation^a^	X	X

^a^Follow-up if necessary

Finally, we attempted to further classify the syndrome according to the involvement of other CNs by identifying the incidence of additional neuro-specific symptoms.

## Results

### Clinical findings

Fifty children were evaluated in our Centre. Among these, 34 satisfied the above mentioned major criteria for MBS diagnosis while 9 patients missing one of the two major criteria were classified as having a Moebius-like syndrome. Five children exhited complex conditions aggravating their clinical expression with Carey-Fineman-Ziter syndrome diagnosed in two, severe myopathy in one, one child had neonatal asphyxia and severe cerebral palsy, and one had psychiatric symptoms. Two patients with a doubtful diagnosis were excluded.

Thirty one percent of the children (15/48) were assessed between 1 to 6 months of age, 15 % between 6 and 12 months of age (7/48), and 21 % between 12 and 36 months of age (10/48). The remaining 16 children (33 %) were older than 3 years. The mean age at the time of the first examination was 38 months.

All cases were sporadic with negative family histories for genetic disorders. Most of these MBS patients were born at term after an uneventful pregnancy. In four cases the pregnancy was complicated by ultrasound evidence of clubfoot and in one case was associated with ventriculomegaly and intrauterine growth retardation. Cerebral magnetic resonance imaging (MRI) was carried out in 16 patients 11 of which were found to be normal. In the remaining five cases, cerebral anomalies such as sixth and seventh CN hypoplasia were evident. Brainstem hypoplasia and bilateral ventricle enlargement was detected in one child and hypophysis hypoplasia was seen in another.

In all cases karyotyping was performed and was normal. Array-CGH was carried out only in cases affected by Carey-Fineman-Ziter syndrome and severe myopathy and the results were normal.

The VII CN (total/partial, bilateral or unilateral) was involved in 96 % of cases and the VI CN in 85 %. Two patients with no impairment of the VII CN and seven patients without involvement of the VI nerve, but with deficiencies of other CNs were defined as being Moebius-like. Other affected CNs were the III-IV in 8 subjects (16 %), the V in 5 (11 %), the VIII and X in 4 (8 %), the XI in 3 (6 %), the IX but usually partially in 10 (22 %) of cases, and the XII in 18/48 (37 %). One case hospitalized in our Neonatal Intensive Care Unit, with involvement of the X CN and calcifications pontine at MRI, died at one month of age due to the absence of respiratory drive. In 19 % of patients Pierre Robin syndrome was evident; 9 % of children showed Poland syndrome; clubfeet were found in 24 %; 11 % showed skeletal anomalies, especially of the hands, feet, or spine (scoliosis); hearing loss or deafness were found in 8 % of children; 8 % were being fed by nasogastric tube until 1 year of age; 5 % demonstrated myopathic features; and 13 % were intellectually disabled.

### Functional findings

Due to the multifactoriality of the disorders, we separated the clinical features according to their functional involvement into the following three major domains as determined by their CN involvement: feeding, language and hearing and visual functions. In the feeding and oral domain (involvement of V, VII, IX, XI, XII CNs) we found poor neonatal sucking and swallowing in 37,8 % of our patients, need for nasogastric tubes and gastrostomy in 5,5 %, nutritional problems in 16 %, dental problems in 17 % and palatal problems or micrognathia in 7,4 %. Problems in language and hearing (involvement of VII, VIII, IX, X, XI, XII CNs) caused hearing loss in 6,8 % of patients, language delay in 31 %, speech deficit in 42 %.

Disturbance of the visual domain (involvement of III, IV, VI, VII CNs) included deficits of both ocular motility and neurovisual function, was evident in 89,8 % and in 19,3 % of children, respectively. Photophobia was also observed in 15 % (Table 4) [18–25].

**Table 4:** Functional symptoms according to specific cranial nerve involvement

Area	Feeding and oral area	Percentage	Language and hearing	Percentage	Vision/visual	Percentage
Cranial nerve involvement	V, VII, IX, XI, XII		VII, VIII, IX, X, XI, XII		III, IV, VI, VII	
Functional Deficit	Poor neonatal sucking and swallowing	37,8	Hearing loss	6,8	Ocular motor deficit	89,8
	Need for nasogastric tubes and gastrostomy	5,5	Language delay	31	Visual deficit	19,3
	Nutritional problems	16	Speech deficit	42	Photophobia	15
	Dental problems	17				
	Palatal problems and micrognathia	7,4				

Three additional important functional areas, neurological, musculoskeletal and cognitive-emotional are also summarized in Table 5. It is noteworthy that neurological findings included hypotonia in 26 % of patients and balance impairment in 12 %. Musculoskeletal involvement included thoracic anomalies observed in 5 %, absence or hypoplasia of pectoralis major muscle in 3 %, chest, arms and hand deformities in 14 %, clubfeet in 32 %, and scoliosis in 6 %. In the cognitive emotional area, we found attention deficit in 17 % of children, cognitive impairment and developmental delay in 15 %, sleep and regulation disturbances in 28 % and stereotypies in 4,2 % (Table 5).

**Table 5:** Affected domains according to body function and structure

Domain	Neurological	Percentage	Musculoskeletal	Percentage	Cognitive-emotional	Percentage
	Hypotonia	26	Thoracic anomalies	5	Attention deficit	17
Functional Deficit	Balance impairment	12	Absence or hypoplasia of pectoralis major muscle	3	Cognitive impairment and developmental delay	15
			Chest, Arms and hand deformities	14	Sleep and regulation disturbances	28
			Club feet	32	Stereotypies	4,2
			Scoliosis	6		

### Neuropsychological findings

Excepting the Moebius-like, the complex cases and the unclassified ones, GMDS-R was carried out in 34 children.

Data showed a delay in the General Quotient (GQ) at 1 year of age (i.e., GQ 82) with a homogeneous profile in all the subscales. At 2 years we noted a modest improvement of the GQ to 89, with delay most evident in motor, behavioral and language subscales. At 3 years of age the mean GQ was 90 with deficiencies in specific cognitive subscales, particularly in hand-eye coordination while at 5 years, the average GQ was 103, with lowest results in the motor subscale due to clumsiness (Table 6).

**Table 6:** Neuropsychological Profile between 1 and 5 years in 34 MBS children

	1 year	2 years	3 years	5 years
**GQ**	**82**	**89**	**90**	**103**
Locomotor	74	67	91	94
Personal social	79	87	97	100
Language	92	86	101	105
Eye and hand coordination	84	90	82	111
Performance	85	90	88	105
Practical reasoning	-	100	98	103

In bold it is shown the general quotient (GQ), while below are listed the values of the 6 subscales

## Discussion

MBS consists of complete or partial facial diplegia, often accompanied by other CN deficiencies.

Most of the previous reviews of MBS tend to focus on the neuroradiological and systemic features of the syndrome, but comprehensive developmental evaluations were not specifically investigated [1–13].

We interpreted the neurofunctional data of the psychometric assessments, taking into account the children’s dysfunctional nutrition (arising from difficulties in feeding), communication and visual-motor function.

The suffering of the children caused by difficulties in management of feeding tubes and the consequent frustration of many of the mothers because they were unable to directly feed their children reduced the pleasure of the food interaction, which plays a large role in building a secure attachment and sense of competency of the mother-child dyad. The lack of facial communicative expressions and the difficulties in speech sounds that negatively affect the reward of the parent-child feedback interaction also can lead to failure of the normal mother-child attachment.

Furthermore, oculomotor dysfunction has serious negative consequences in motor, perceptual and cognitive development. The visual system, provides the function of the "what", the recognition of objects and shapes, but the ocular movements provide the functions of the "where", that is, the location of objects, providing data on spatial orientation, the perception of movement and the third dimension (across saccades, optokinetic nystagmus, smooth pursuit, scanning and visual exploration).

Our experience has suggested that MBS children exhibit not only the above main morpho-functional deficits involving movement, food, vision and language, but also secondary developmental problems.

Primary motor problems consisted of hypotonia, musculo-skeletal dysmorphism, delay in righting, locomotion and clumsiness. Feeding disorders consisted of sucking-swallowing difficulty, breathing problems, lack of the sensitivity of the orofacial area, dysphagia with the need, in severe cases, for a gastrostomy. Language difficulties such as dyslalia, mechanical disorders of phonological coding and speech structure and writing were common. Visual impairment consisted of scanning, exploration and visual-perceptual deficits, and, in some cases, corneal ulcers.

Secondary problems included visual exploration deficits, oral-motor difficulties as well as lack of the categorization of facial expressions affecting cognitive strategies in early development. Pain and other difficulties during eating, maternal concerns, and lack of recognition of emotions, can form the basis of significant emotional distress. From the perspective of social maturity, self-image and communication with peers can be affected adversely both in childhood and in adolescence.

Equally important is the peculiar functional profile of MBS children. In general, they usually present with delay at 1 year of age, with motor emotional and language difficulties persisting until 2 years of age and reduced hand-eye coordination at 3 years of age. Children generally achieve average levels of GQ at five years of age although with retained aspects of clumsiness. Overall 90 % of children reached a normal GQ with only 10 % maintaining cognitive deficits.

The gradual recovery in fragile areas and the progressive harmonization of functions might be explained by early rehabilitation and by support by and of parents. Improving the methods of early intervention, enhancing motor ability and sensory information, and the timely support of parents having emotional difficulties, can improve the prognosis of the child with MBS.

**Fig. 1:**
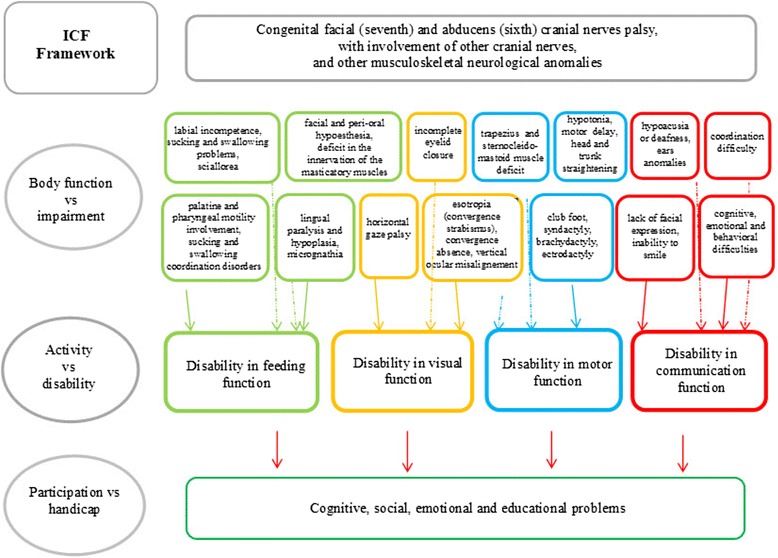
MBS’ Interpretation ICF

For all of these reasons, the International Classification of functioning Children and Youth (ICF-CY), published by the WHO can be considered the gold standard for describing functioning, disability and health. It appears to be a suitable framework for interpreting functions (vs impairment), activities (vs disability) and participation (vs handicap), in children with MBS and for evaluation and intervention of the familial and social environment [26]. In particular, the ICF-CY framework allows the health care practitioners to investigate and integrate the complexity of signs and functions in children with MBS such as motor skills (musculoskeletal features, posture, posture changes, gait and coordination), feeding (sucking patterns, quality of food, eating habits, sucking-swallowing coordination), communication and language (phonological, articulatory, oral motor skills and functional aspects), and cognitive features. The frequency and uniqueness of medical problems found in patients with MBS suggest the need for such a protocol to carefully define guidelines for the current and continuing care of these patients (Fig. 1).

## Conclusion

The main finding of this study is the observation that the patients with MBS exhibit development characterized by global delay at 1 year of age, motor, emotional and speech difficulties at 2 years of age, a trend toward normalization at 3 years of age but with weakness in hand-eye coordination, and finally achieving average results at 5 years of age.

It is important that diagnosis and rehabilitation start simultaneously and that the rehabilitative intervention is updated over time in response to functional assessments. It is also essential that any intervention address both the child and the mother-child relationship. The approach must also be multidisciplinary, involving professionals with different skills and with specific training in cooperative therapies.

Lastly, the analyzed population requires larger numbers and needs a more prolonged follow-up to assess the developmental outcomes and the complexity of these children.
